# Epithelial to mesenchymal transition in human endocrine islet cells

**DOI:** 10.1371/journal.pone.0191104

**Published:** 2018-01-23

**Authors:** José Luis Moreno-Amador, Noèlia Téllez, Sandra Marin, Caterina Aloy-Reverté, Carlos Semino, Montserrat Nacher, Eduard Montanya

**Affiliations:** 1 Institut d’Investigació Biomèdica de Bellvitge (IDIBELL), Barcelona, Spain; 2 CIBER de Diabetes y Enfermedades Metabólicas Asociadas (CIBERDEM), Madrid, Spain; 3 IQS School of Engineering, Universitat Ramon Llull, Barcelona, Spain; 4 Hospital Universitari Bellvitge, Hospitalet de Llobregat, Spain; 5 University of Barcelona, Barcelona, Spain; University of Alabama at Birmingham, UNITED STATES

## Abstract

**Background:**

β-cells undergo an epithelial to mesenchymal transition (EMT) when expanded in monolayer culture and give rise to highly proliferative mesenchymal cells that retain the potential to re-differentiate into insulin-producing cells.

**Objective:**

To investigate whether EMT takes place in the endocrine non-β cells of human islets.

**Methodology:**

Human islets isolated from 12 multiorgan donors were dissociated into single cells, purified by magnetic cell sorting, and cultured in monolayer.

**Results:**

Co-expression of insulin and the mesenchymal marker vimentin was identified within the first passage (p1) and increased subsequently (insulin^+^vimentin^+^ 7.2±6% at p1; 43±15% at p4). The endocrine non-β-cells did also co-express vimentin (glucagon^+^vimentin^+^ 59±1.5% and 93±6%, somatostatin^+^vimentin^+^ 16±9.4% and 90±10% at p1 and p4 respectively; PP^+^vimentin^+^ 74±14% at p1; 88±12% at p2). The percentage of cells expressing only endocrine markers was progressively reduced (0.6±0.2% insulin^+^, 0.2±0.1% glucagon^+^, and 0.3±0.2% somatostatin^+^ cells at p4, and 0.7±0.3% PP^+^ cells at p2. Changes in gene expression were also indicated of EMT, with reduced expression of endocrine markers and the epithelial marker *CDH-1* (p<0.01), and increased expression of mesenchymal markers (*CDH-2*, *SNAI2*, *ZEB1*, *ZEB2*, *VIM*, *NT5E* and *ACTA2*; p<0.05). Treatment with the EMT inhibitor A83-01 significantly reduced the percentage of co-expressing cells and preserved the expression of endocrine markers.

**Conclusions:**

In adult human islets, all four endocrine islet cell types undergo EMT when islet cells are expanded in monolayer conditions. The presence of EMT in all islet endocrine cells could be relevant to design of strategies aiming to re-differentiate the expanded islet cells towards a β-cell phenotype.

## Introduction

Cell therapy of diabetes is limited by the shortage of β-cells available for transplantation. *In vitro* expansion of functional human β-cells is an attractive possibility to generate an abundant source of insulin-producing cells. Adult β-cells have a low replicative capacity, but when cultured in monolayer they undergo a phenotypic shift through an epithelial to mesenchymal transition (EMT) process and give rise to highly proliferative mesenchymal cells that can be massively expanded [1,2]. These expanded cells retain the potential to re-differentiate into insulin-producing cells [3]. Since EMT has been identified in other human epithelial cells cultured in 2D systems [4], we hypothesized that it could take place as well in the endocrine non-β cells of the islets when expanded *in vitro*. The presence of EMT in the endocrine non-β cells could have implications for the design of strategies aiming to generate new sources of insulin producing cells, particularly in the light of the transdifferentiation potential of α and δ-cells into β-cell like cells [5]. However, EMT has not been directly investigated in the endocrine non-β cells of the islets. Loss of glucagon expression has been described in expanded islet cells, but it was not determined whether it was related to EMT, and contrary to insulin expression, somatostatin and PP expression was maintained after several passages in culture [2], suggesting the possibility that δ and PP cells do not undergo EMT or they do it at a lower rate. Thus, we aimed to determine whether EMT takes place in the endocrine non-β-cells of human pancreatic islets expanded *in vitro*.

## Material and methods

### Human islet cell isolation

Pancreatic islets were isolated from 12 adult cadaveric organ donors (age 54±5 y.o and BMI 26±1 kg/m^2^) by collagenase digestion (Collagenase NB1 Premium Grade with Neutral Protease NB, Serva Electrophoresis GmbH, Heidelberg, Germany) using the Ricordi method with some modifications [6,7]. The islets were purified on a refrigerated COBE 2991 cell processor (COBE BCT, Lakewood, CO, USA) with a continuous density gradient. Immediately after purification, islets were seeded in non-adherent platelet culture bags (Fenwal Europe sprl, Mont Saint Guibert, Belgium) and cultured in CMRL 1066 medium (Connaught Medical Research Laboratories, Mediatech, Inc, Corning Cellgro, Manassas, VA, USA) containing 5.6 mM glucose and supplemented with 10% ABO compatible human serum (Blood and Tissue Bank, BST, Barcelona, Spain) as previously described [7]. The islets were maintained at 37°C and 5% CO_2_ in a humidified incubator for 1–4 days until dissociated into single cells and sorted. Culture medium was changed every two days. Islet purity was determined by dithizone staining (Sigma-Aldrich, St. Louis, MO, USA). The study was approved by the Ethics Committee of Hospital Universitari Bellvitge and written informed consent was obtained from the relatives of organ donors.

### Cell sorting and culture

Islets were dissociated into single cells with 0.16 mg/ml trypsin and 0,1 mmol/l EDTA [8] and filtered through a 40 μm nylon mesh (BD Falcon, Bedford, MA, USA). Once dispersed, single endocrine cells were purified by magnetic activated cell sorting (MACS) as previously described [9]. PSA-NCAM antibody (Miltenyi Biotech, Auburn, CA, USA) was used as a positive selection marker of endocrine cells. Dispersed islet cells were kept for 10 minutes at 4°C in MACS buffer (PBS1X, BSA 0,5%, EDTA 2mM) and incubated with microbeads conjugated to PSA-NCAM antibody for 15 minutes at 4°C. Cell sorting was carried out on a MiniMACS magnetic cell separation system according to manufacturer’s instructions (Miltenyi Biotech). Both PSA-NCAM-positive and negative fractions were seeded for monolayer culture in 35x10 mm tissue culture dishes (Cell+, Sarstedt) at 2x10^4^ cells/cm^2^ density. The medium used was CMRL with 5.6 mM glucose supplemented with 2 mM L-Glutamine (Life Technologies, Thermo Fisher Scientific Inc, MA, USA), 10 mM HEPES (Biological Industries, Kibbutz Beit Haemek, Israel), and with 10% of fetal bovine serum (FBS) (Gibco™, Life Technologies, Carlsbad, CA, USA). Cells were maintained at 37°C in a humidified incubator with 5% CO_2_ [7]. The medium was changed twice a week, and after the initial 12 days in culture (passage 1) the expanded population split 1:2 once a week using 0.25% trypsin-EDTA (Sigma-Aldrich, St. Louis, MO, USA). Cell samples were taken at the end of each passage up to passage 4 (p1 to p4). The PSA-NCAM-positive fraction was used to study the evolution of endocrine populations through passages and to characterize the EMT process by gene expression and immunofluorescence. The PSA-NCAM negative fractions were used to characterize in detail p1 events (days 0, 4, 8 and 12), and to quantify β-cell death.

### Immunostaining and image analysis

At the pre-specified culture times, cells were treated with trypsin (Sigma-Aldrich), harvested, pelleted, fixed with 4% paraformaldehyde (Merck KGaA, Darmstadt, Germany) and processed for paraffin embedding. Pancreas blocks from 5 donors were also fixed with 4% paraformaldehyde (Merck KGaA) and embedded in paraffin. Antigen retrieval was performed using a microwave in 10mM citrate buffer (pH 6.0) and/or trypsin. Cells were blocked with 5% Horse Serum and incubated overnight at 4°C with the primary antibodies (S1 Table). Alexa fluor-conjugated donkey anti-rabbit, goat anti-chicken and goat anti-mouse (1:400; all from Invitrogen, Van Allen Way Carlsbad, CA, USA) were used as secondary antibodies. Images of the double-labeled sections were acquired using a Leica DFC 310FX and Leica TCS-SL filter-free spectral confocal laser scanning microscope (Leica Microsystems, Mannheim, Germany) and processed with the assistance of the ImageJ analytical software (National Institutes of Health, Maryland, U.S.A). Nuclei were stained with 4'-6-diamidino-2-phenylindole (DAPI 300nM) (Life Technologies). Co-expression of mesenchymal marker vimentin and islet endocrine hormones was used to evaluate EMT. Results are expressed as percentage of double-positive cells over the total number of the specific endocrine cell type. A minimum of 500 positive-endocrine cells was counted per sample in at least 5 different random fields.

### β-cell death

Cells were harvested on days 0, 4, 8, 12 of p1 and at the end of p2, pelleted and processed for immunostaining as described. The sections were double-stained for cell death with the TUNEL technique (In Situ Cell Death Detection Kit, ApopTag, Intergene, Merck Millipore, MA, USA), and for insulin with a rabbit polyclonal anti-insulin antibody (Santa Cruz Biotechnology Inc, Texas, USA) (1:100). For insulin labeling, the secondary antibody was a donkey anti-rabbit IgG (Alexa Fluor 555; Life Technologies; 1:400). Nuclei were stained with DAPI 300nM (Life Technologies). Images were taken using a Leica DFC 310FX and processed with the assistance of the ImageJ analytical software. β-cell death was expressed as percentage of TUNEL-positive β-cells. A minimum of 1000 insulin-positive cells were counted per sample.

### EMT inhibition

For EMT inhibition experiments, sorted cells were plated in 35x10 mm tissue culture dishes (Cell+, Sarstedt) at 4x10^4^ cells/cm^2^ density, and seeded for monolayer culture up to 12 days in CMRL medium supplemented as described above. To inhibit EMT, 1 μM of A83-01, a selective inhibitor of TGF-β type I receptor ALK5 kinase (Tocris Bioscience, Bristol, UK), was added to culture medium from day 4 to day 12. Non-treated cells were used as control. Culture medium was replaced everyday in both control and A83-01 conditions.

### RT-PCR analysis

Total RNA extraction was performed using the RNAeasy Plus Minikit (Qiagen, Germany) according to the manufacturer’s instructions. RNA quantity and quality (RIN ≥ 7 in all samples) was measured using a 2100 Bioanalyzer (Agilent Technologies, Palo Alto, CA). cDNA was synthesized using High Capacity cDNA RT Kit (Applied Biosystems, Foster City, CA, USA) followed by RNaseH treatment (Invitrogen). qPCR reaction was carried out in triplicates using TaqMan Universal PCR Master Mix combined with TaqMan chemistry in 7900 Real-Time PCR System (Applied Biosystems). Analysis of relative gene expression was calculated with the 2^-*ΔΔCt*^ method [10] and using human TATA-box binding protein (TBP) and human large ribosomal protein (RPLP0) as endogenous controls. Data were analyzed using Expression Suite Software v1.0.3. Full listing of assays (Applied Biosystems), gene names and assay identification numbers is given in S2 Table. Reactions were performed according to manufacturer’s instructions. Cycle number 40 was used for undetectable transcripts. Relative quantity values were normalized to give a mean of 1 for control (day 0) to aid in comparison across genes with varying basal abundance.

### Statistical analysis

Statistical analysis was performed GraphPad Prism 5.0 (GraphPad, La Jolla, CA, USA. Results are expressed as means ± SEM. Data were analyzed using Student’s *t*-test, One-way ANOVA combined with Tukey’s test for post-hoc analysis or Kruskal-Wallis one-way analysis combined with the post-hoc Dunn’s test for multiple comparisons as appropriate. A *P* value < 0.05 was considered statistically significant.

## Results

### Cell purification

**Fig 1:**
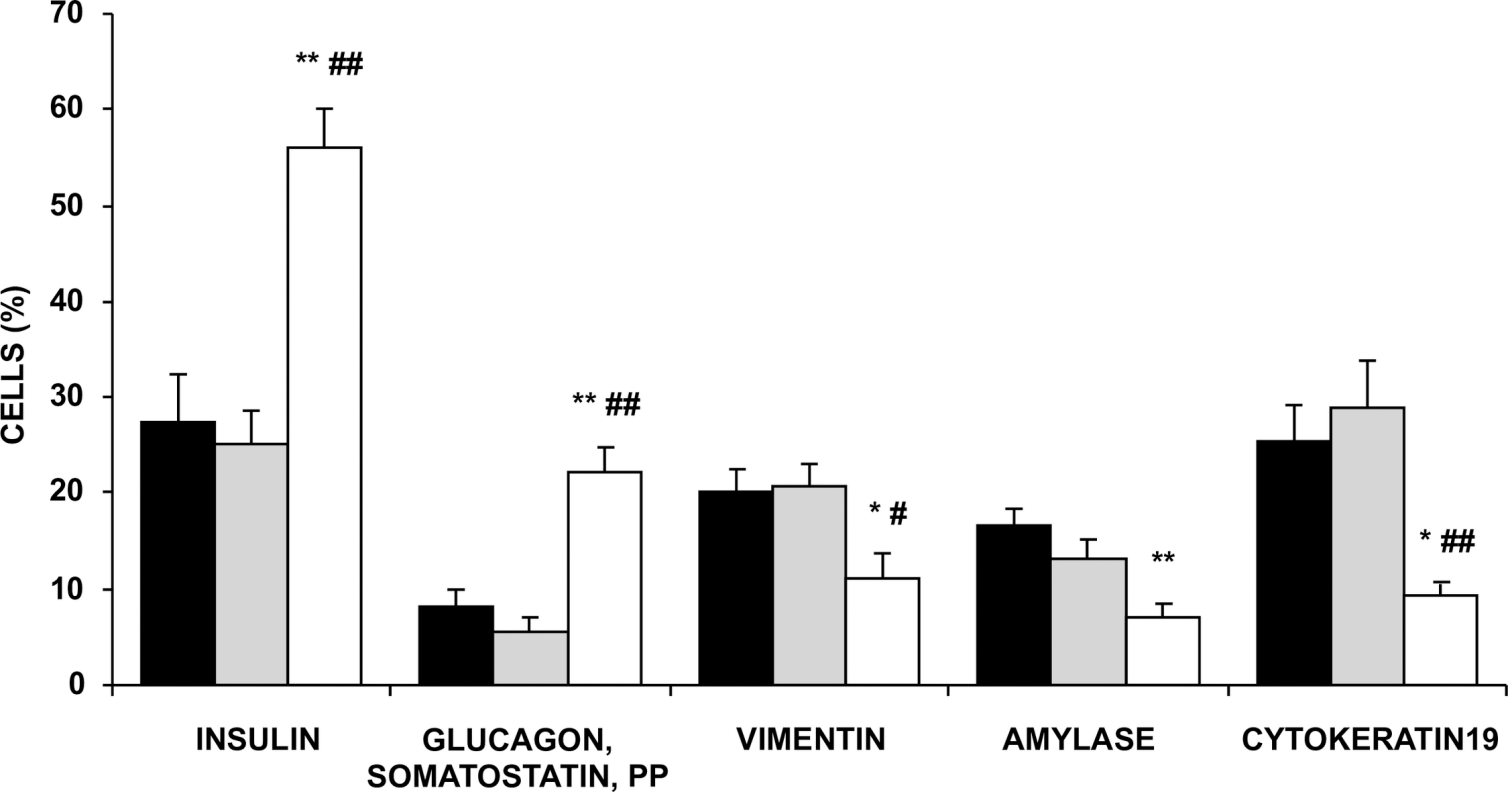
Purification of pancreatic endocrine cells. Cellular composition of pre-sorting preparations (black bars), and PSA-NCAM negative (grey bars) and positive (white bars) fractions. Data are means ± SEM (n = 8). ANOVA, P< 0.05 with post-hoc Tukey’s test for multiple comparisons, * P< 0.05 and ** P< 0.01 vs pre-sorting; ^**#**^ P< 0.05 and ^**##**^ P< 0.01 vs PSA-NCAM negative fraction.

After islet isolation, the cell preparations were dispersed into single cells and sorted by MACS to further increase the endocrine cell purity. Magnetic cell sorting resulted in a significant enrichment in insulin^+^ cells in the PSA-NCAM-positive fraction (pre-sorting: 27 ± 5%, post-sorting: 56 ± 4%), and in endocrine non-β-cells (pre-sorting: 8 ± 2%, post-sorting 22 ± 3%) (Fig 1). Thus, the endocrine cell purity in the post-sorting fraction was 78 ± 4%. The presence of amylase^+^ and cytokeratin 19^+^ (Ck19^+^) cells, as well as vimentin^+^ cells, was significantly reduced in the PSA-NCAM positive post-sorting fraction.

### Changes in cell phenotype along culture passages

**Fig 2:**
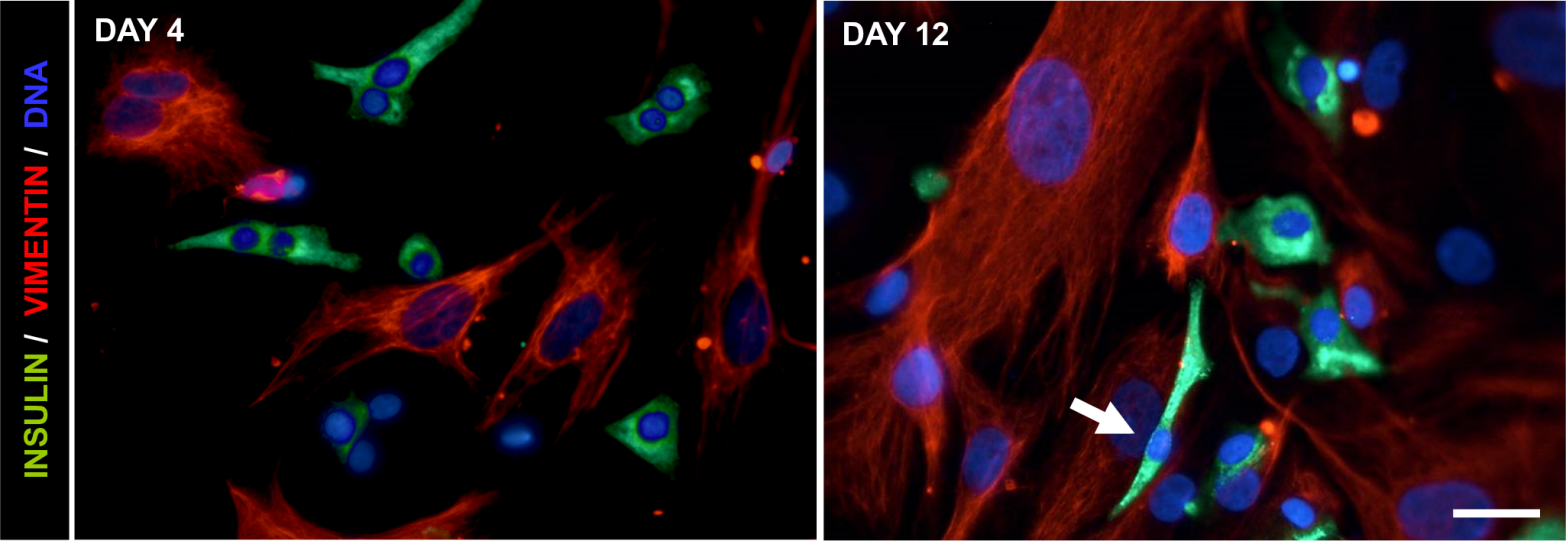
Phenotypic evolution of expanded β-cells. Representative immunofluorescence images of day 4 and day 12 cell preparations stained with insulin (green) and vimentin (red) showing the acquisition of a fibroblast-like phenotype by insulin-positive cells (arrow). Scale Bar = 20μm.

After 4 days in monolayer culture, the endocrine cells maintained their characteristic epithelial morphology, but at the end of passage 1 (day 12) most cells showed a fibroblast-like phenotype (Fig 2).

**Fig 3:**
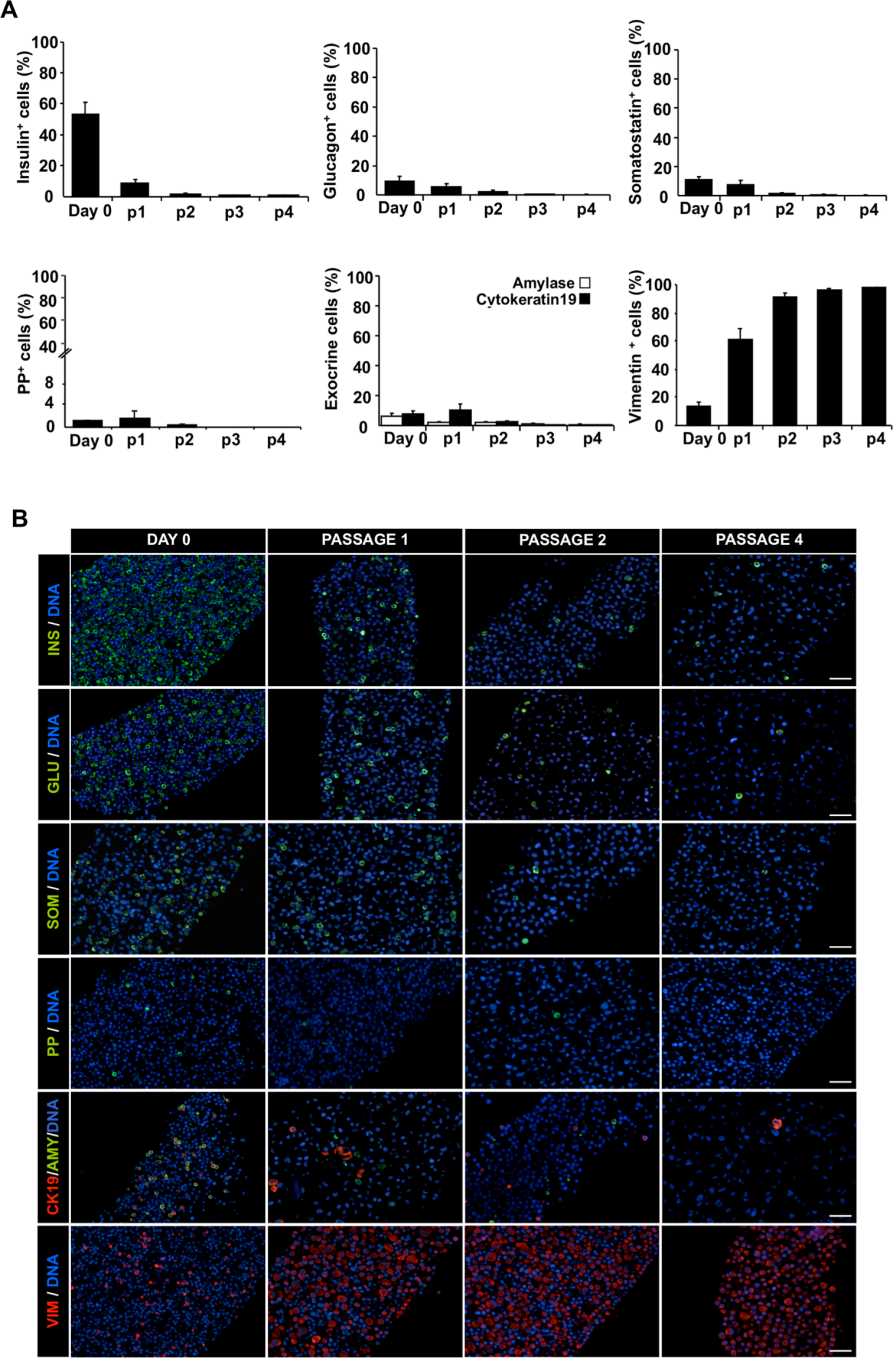
Loss of pancreatic endocrine and exocrine markers during *in vitro* expansion. (A) Cell composition along passages. A minimum of 500 positive cells for each population was counted on at least five representative fields. Data are means ± SEM (n = 6). (B) Representative immunofluorescence images of cell preparations on day 0, and passages 1 to 4 stained for the pancreatic endocrine hormones insulin, glucagon, somatostatin and pancreatic polypeptide (green), the exocrine markers amylase (green) and cytokeratin19 (red), and the mesenchymal marker vimentin (red). Nuclei are stained in blue with DAPI. Scale Bar = 50μm.

The percentage of insulin^+^ cells decreased from 53.4 ± 7.3% (day 0) to 8.5 ± 1.9% (day 12), and they were almost undetectable at p4 (0.6 ± 0.2%) (Fig 3). The percentage of glucagon^+^ cells (day 0: 9.5 ± 3.3%; day 12: 5.6 ± 2.4%,), and somatostatin^+^ cells (day 0: 10.8 ± 2.0%; day 12: 7.3 ± 3.0%) was also reduced, even though less dramatically than insulin^+^ cells, and they were not identified beyond p4. Pancreatic polypeptide^+^ cells were scarce on day 0 (0.9 ± 0.2%) and were not detected beyond p2. The percentage of cells expressing the exocrine markers Ck19 and amylase decreased from 7.6 ± 2.2% to 0.4 ± 0.2% and from 6.4 ± 1.6% to 0.7 ± 0.3%, respectively (day 0 and p4). In contrast, the percentage of vimentin^+^ cells increased from 13.5 ± 2.9% on day 0 to 60.7 ± 7.9% on day 12, and to 98.0 ± 0.5% at p4.

**Fig 4:**
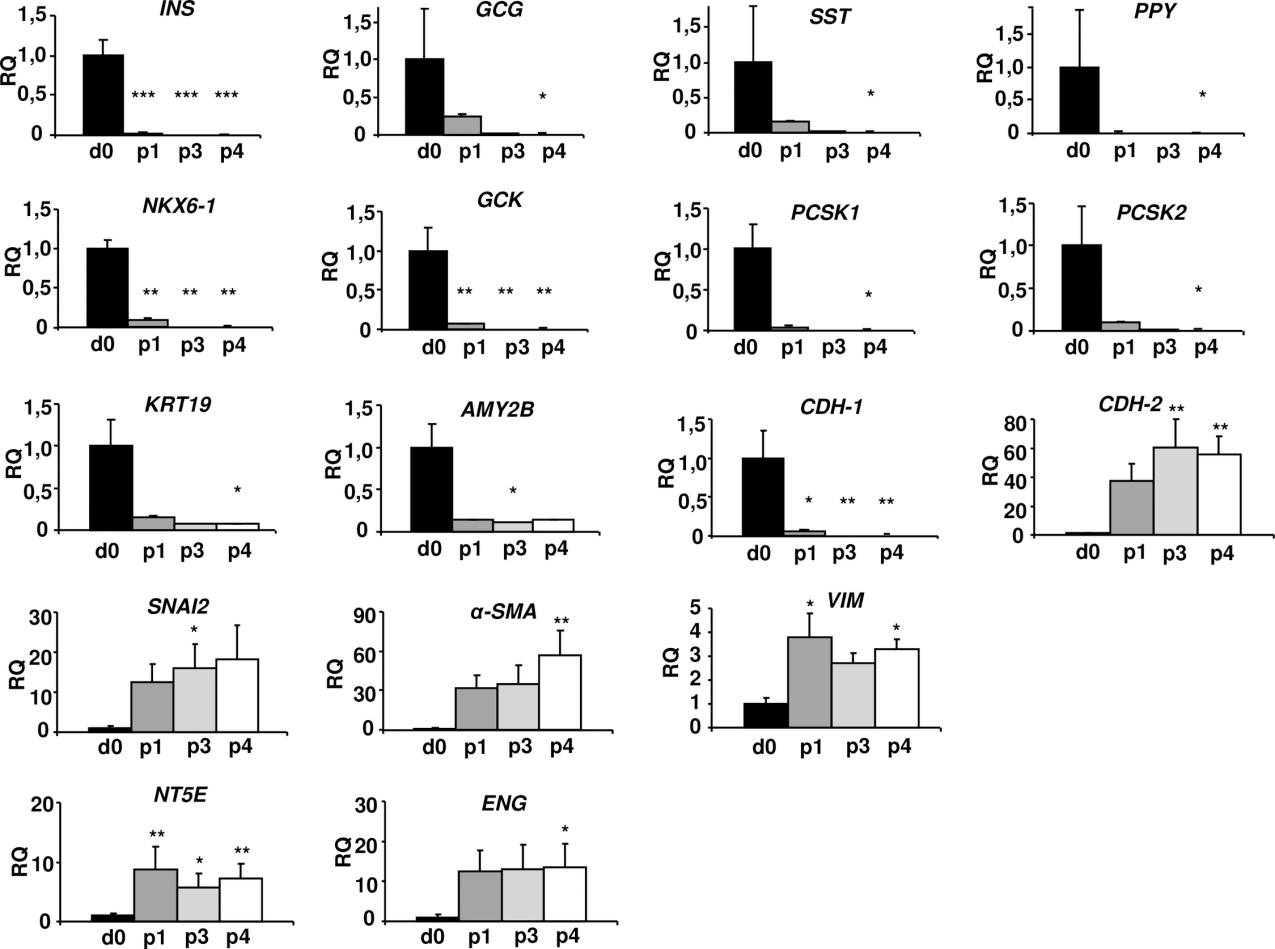
Changes in the expression of epithelial and mesenchymal genes during *in vitro* expansion. Gene expression of pancreatic islet hormones, other endocrine markers, exocrine markers and epithelial marker E-cadherin (*CDH-1)* was progressively reduced. The expression of the EMT transcription factor *SNAI2* and other mesenchymal markers was increased. RQ: relative mRNA levels. Data are means ± SEM (n = 5). ANOVA, P< 0.05 with post-hoc Tukey’s test or Kruskal-Wallis one-way analysis combined with the post-hoc Dunn’s test, for multiple comparisons. * P< 0.05; ** P< 0.01; *** P< 0.0001 vs day 0 (d0).

Gene expression of insulin *(INS)*, glucagon *(GCG)*, somatostatin *(SST)* and PP *(PPY)* was markedly reduced at the end of p1 and became almost undetectable at p4 (Fig 4). The expression of the β-cell transcription factor *NKX6*.*1*, the enzyme glucokinase (*GCK*), and the prohormone convertases *PCSK1* and *PCSK2*, was similarly reduced. The expression of the pancreatic exocrine markers Ck19 (*KRT19*) and amylase (*AMY2B*) was markedly reduced at the end of p1. The expression of the epithelial marker E-cadherin (*CDH-1)*, the hallmark of the epithelial state, was significantly reduced at p1, and it then became almost undetectable. Loss of expression of *CDH-1* is considered an early indication of EMT [11]. Conversely, we identified an up-regulation of transcripts encoding the mesenchymal markers vimentin (*VIM*), smooth-muscle actin (*α-SMA*), and N-cadherin (*CDH-2*), the mesenchymal stromal cells (MSC) markers ecto-5'-nucleotidase (*NT5E)* and endoglin (*ENG)*, and the EMT-associated marker *SNAI2* (Fig 4), suggesting also the presence of an EMT process in cultured cells.

### Changes in cell phenotype on passage 1

**Fig 5:**
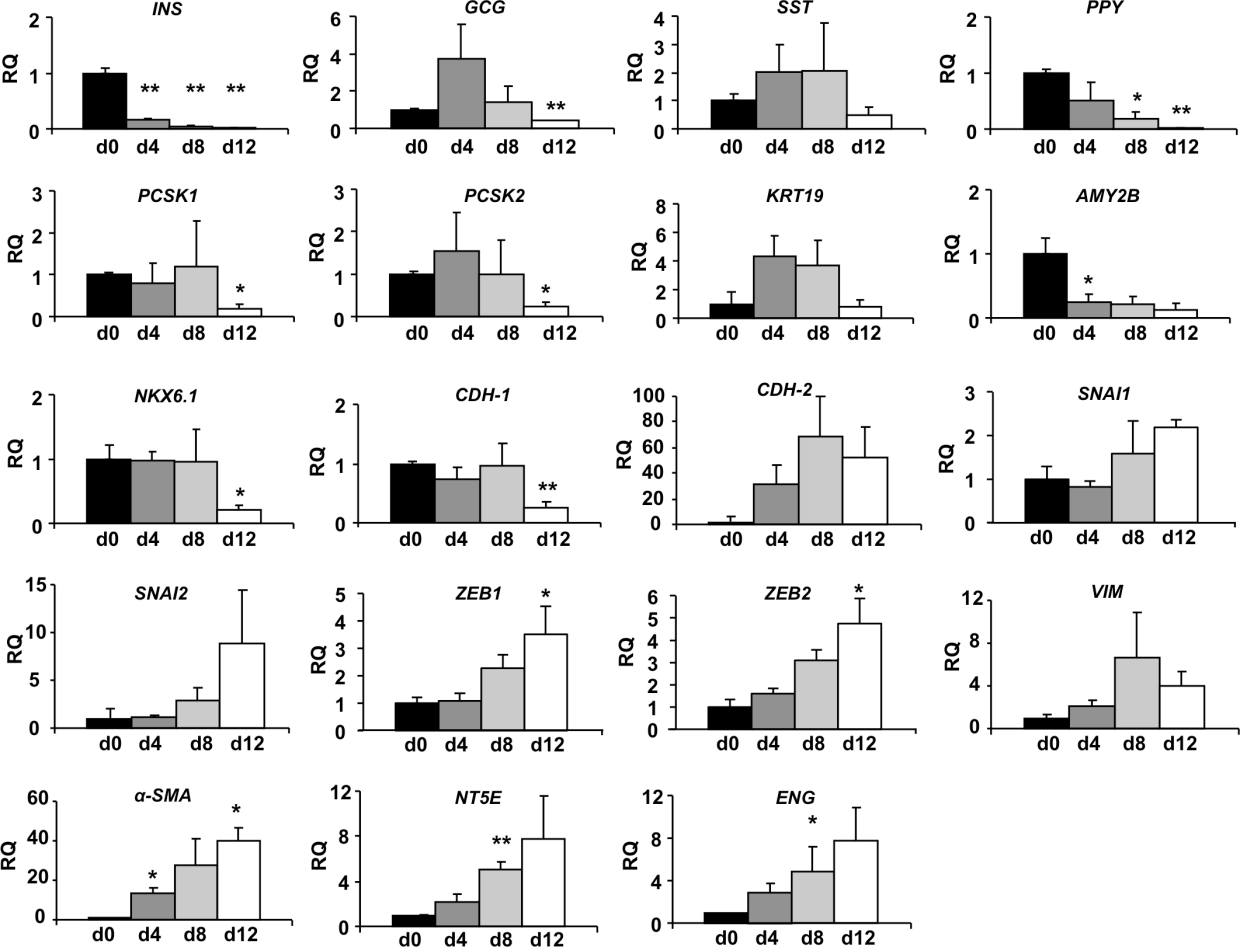
Changes in gene expression at passage 1. Gene expression analysis of the initial p1 period (days 0, 4, 8 and 12). Results are expressed as relative mRNA levels (RQ). Data are means ± SEM (n = 3–4 donors). * P< 0.05 and ** P< 0.01 vs day 0.

Since the most dramatic changes in cell composition took place in p1, we performed a more detailed analysis of cell characteristics on culture days 0, 4, 8 and 12. *INS* gene expression was significantly reduced on day 4 (Fig 5). In contrast, *GCG* gene expression tended to increase on day 4 and was not significantly reduced until day 12.

**Fig 6:**
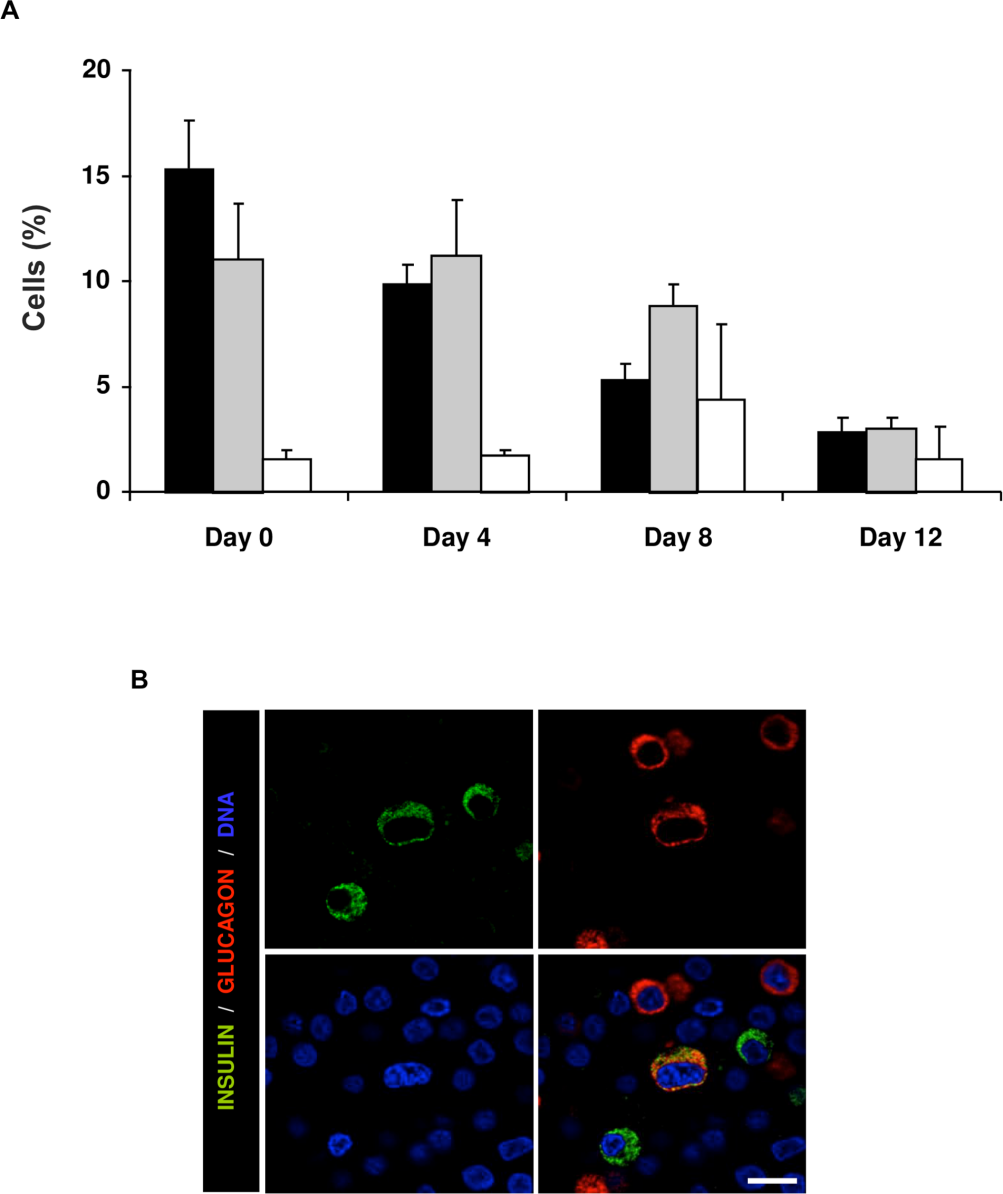
Insulin/glucagon double positive cells. (A) Percentage of β (black bars), α (grey bars) and double positive cells (white bars) along passage 1 in non-enriched PSA-NCAM-negative fractions. A minimum of 500 positive cells per sample was counted. Data are expressed as means ± SEM (n = 3 donors). (B) Representative confocal immunofluorescence images showing co-expression of insulin (green) and glucagon (red). Scale bar = 20 μm.

Concordantly, the percentage of cells expressing insulin was reduced on day 4, and declined progressively along p1, while the percentage of glucagon positive cells remained stable until day 12 (Fig 6A). To explore in more detail this divergent evolution, we determined the presence of insulin^+^/glucagon^+^ double positive cells (Fig 6B). On day 0, 1.5 ± 0.4% of insulin^+^ cells were also glucagon^+^, and the percentage increased transiently (day 4: 1.7 ± 0.6%; day 8: 4.4 ± 3.6%; day 12: 1.6 ± 1.5%) (Fig 6A). The increased percentage of double-positive cells, along with the divergent evolution of *INS* and *GCG* expression suggests the presence of a β to α transdifferentiation.

The initial evolution of acinar and ductal cell markers *KRT19* and *AMY2B* was also divergent. The expression of *AMY2B*, was significantly reduced (p = 0.02) while *KRT19* tended to increase (p = 0.08) on day 4 (Fig 5). This is in agreement with the described transdifferentiation of acinar cells into ductal cells during *in vitro* expansion [12,13]. The expression of the EMT markers *ZEB1* (p = 0.045) *and ZEB2* (p = 0.036), and the MSC markers increased progressively indicating the early initiation of EMT.

### β-cell death

**Fig 7:**
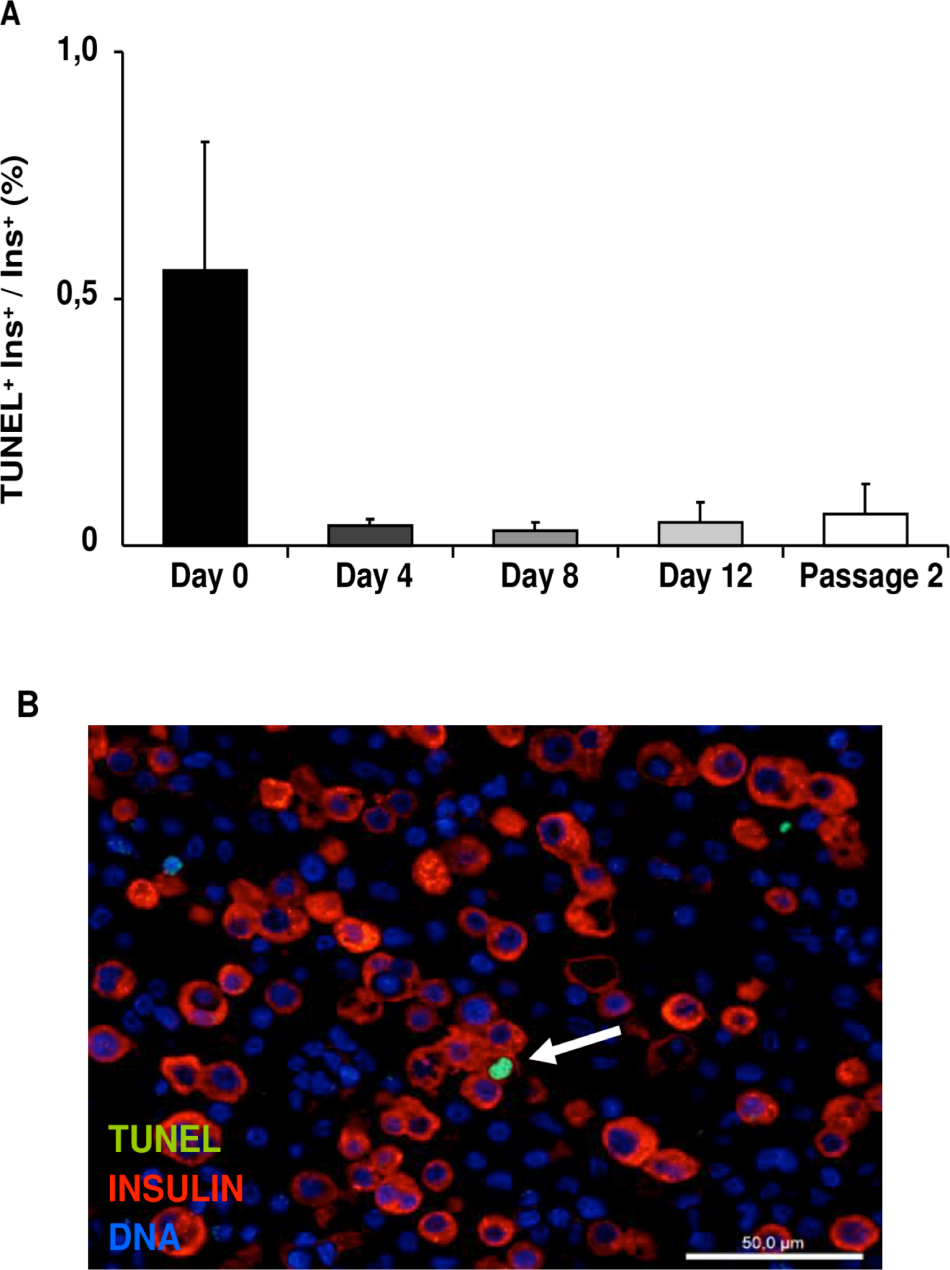
β-cell death. (A) β-cell death in passage 1 (days 0, 4, 8 and 12) and passage 2. A minimum of 1000 β-cells per sample was counted. Data are means ± SEM (n = 6). (B) Representative immunofluorescence image showing double staining for TUNEL (green) and insulin (red) on day 12. Nuclei are stained in blue with DAPI. Arrow indicates a TUNEL^+^/insulin^+^ β-cell. Scale bar = 50μm.

The percentage of dead β-cells (TUNEL^+^/insulin^+^) was low after MACS purification, (day 0: 0.56 ± 0.26%), and it was even lower (between 0.03% and 0.06%) on days 4, 8, 12 of culture as well as at the end of p2 (Fig 7), indicating that the reduction in insulin-expressing cells in p1 and p2 was not due to increased β-cell death.

### EMT in endocrine cells

**Fig 8:**
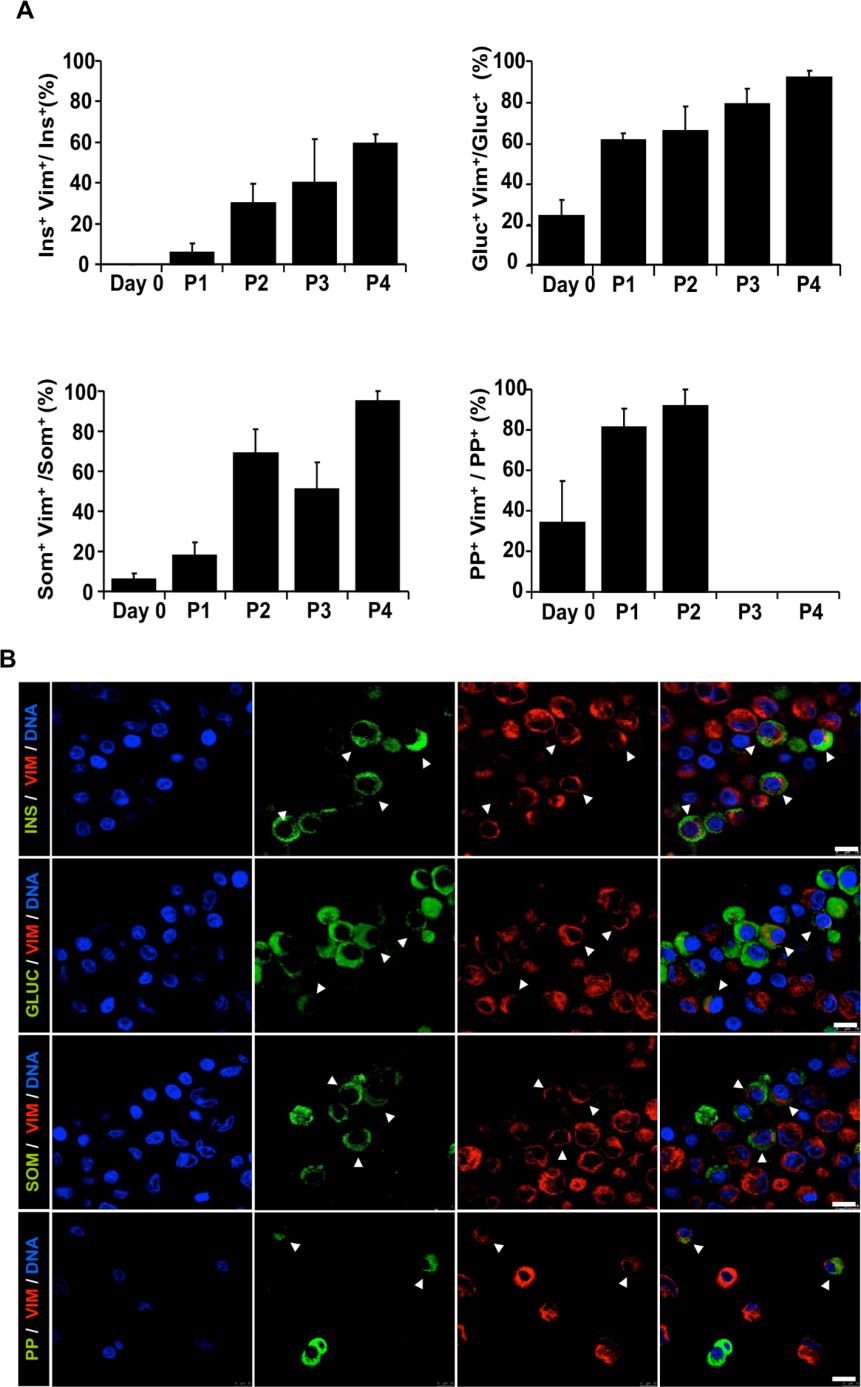
Epithelial to mesenchymal transition (EMT) in endocrine cells. (A) Percentage of double positive cells (endocrine/vimentin marker) in each passage. PP positive cells were not detected beyond P2. A minimum of 500 positive cells was counted for each cell type in a minimum of 5 representative fields. Data are means ± SEM (n = 6). (B) Representative confocal immunofluorescence images of cells at p1 stained with the specified endocrine marker (green) and vimentin (red). Nuclei are stained in blue with DAPI. Arrows indicate endocrine/vimentin double positive cells undergoing EMT. Scale bar = 10μm.

To investigate the presence of EMT in endocrine cells, we determined the co-expression of the mesenchymal marker vimentin in cells expressing insulin, glucagon, somatostatin or PP. On day 0, the expression of the vimentin was detected in glucagon^+^ (24.7 ± 7.6%), somatostatin^+^ (6.0 ± 2.3%), and PP^+^ (33.9 ± 20.9%) cells, but was rare in insulin^+^ cells (0.1 ± 0.1%, detectable in only 2 of the 12 preparations) (Fig 8). After 4 days in culture co-expression of insulin and vimentin was detected in 1.7 ± 0.9% insulin^+^ cells, and increased to 5.4 ± 4.9% at the end of p1 and to 59.2 ± 4.4% at p4. Co-expression of somatostatin and vimentin increased from 17.9 ± 6.4% to 95 ± 5% of somatostatin^+^ cells (p1 and p4 respectively); co-expression of glucagon and vimentin increased from 61.7 ± 3.7% at p1 to 92.9 ± 4.1% of glucagon^+^ cells at p4, and co-expression of PP^+^ cells and vimentin from 33.9 ± 20.9% on day 0, to 80.9 ± 9.4% and 91.7 ± 7.3%, at the end of p1 and at p2 respectively. No PP^+^ cells were identified at p3 and p4. The number of cells counted at each time point, and the percentage of double-positive cells is shown in S3 Table.

### Co-expression of endocrine and mesenchymal markers in the human pancreas

**Fig 9:**
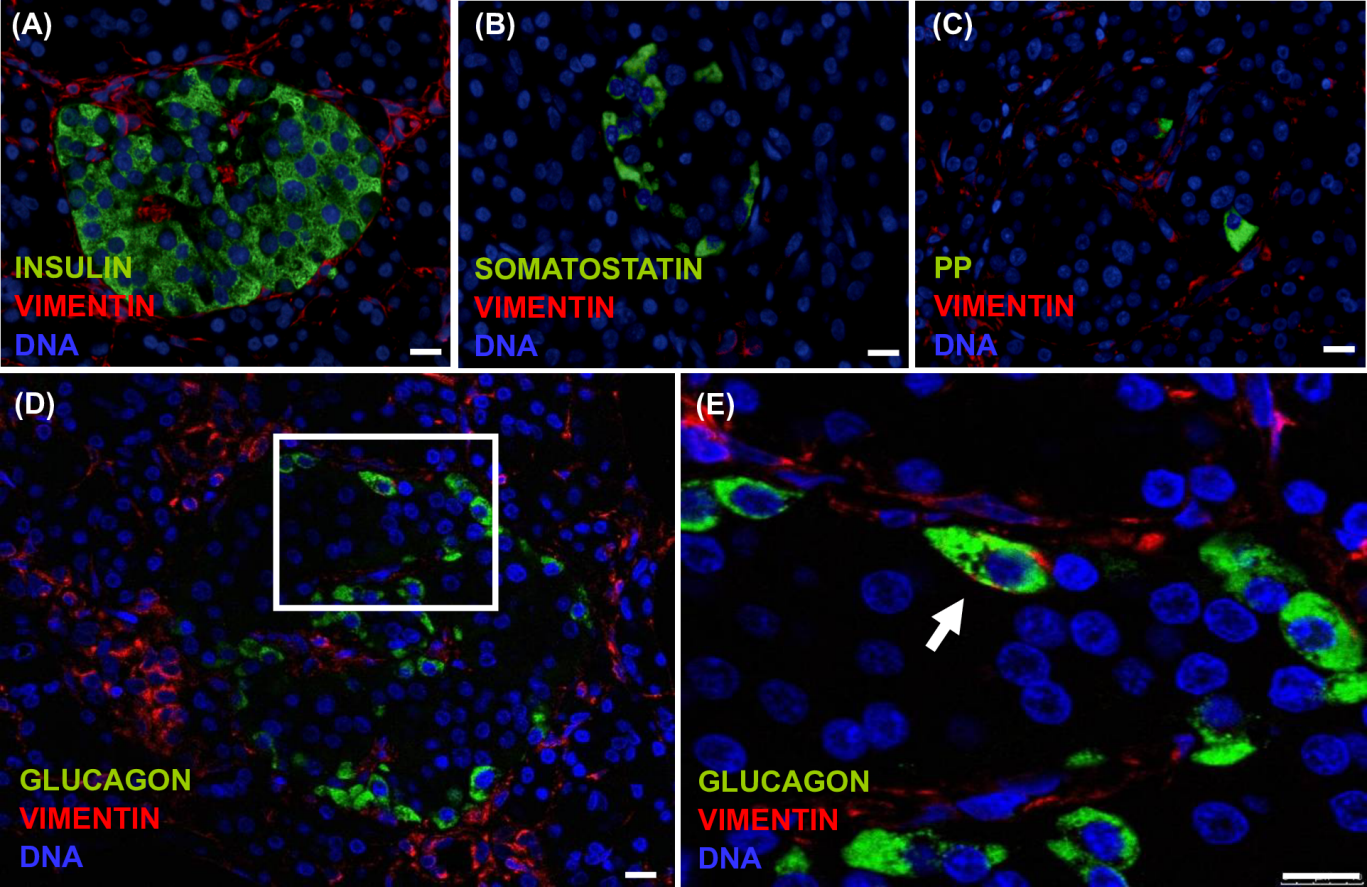
Co-expression of endocrine and mesenchymal markers in the human pancreas. Representative confocal immunofluorescence images of human pancreas sections double stained for the specified endocrine marker (green) and vimentin (red) and). (A-C), β-, δ- and PP-cells were not found to co-express vimentin. (D-E), Islet α-cells co-expressing vimentin (inset) were identified in all pancreas. Scale bar = 20 μm.

The presence on day 0, immediately after islet dispersion into single cells, of cells co-expressing mesenchymal and endocrine markers suggested that the co-expression could already be present in the donor pancreas. To address this question we evaluated sections from the pancreas of 5 non-diabetic donors (age 46 ± 18 y.o, BMI 28 ± 2 kg/m^2^). Co-expression of endocrine hormones and vimentin was found in a low percentage of α-cells (2.8 ± 0.9%), but not in β, δ or PP cells **(**Fig 9), indicating that the expression of vimentin in β, δ and PP cells begins after islet cell isolation and dispersion into single cells.

### EMT inhibition

**Fig 10:**
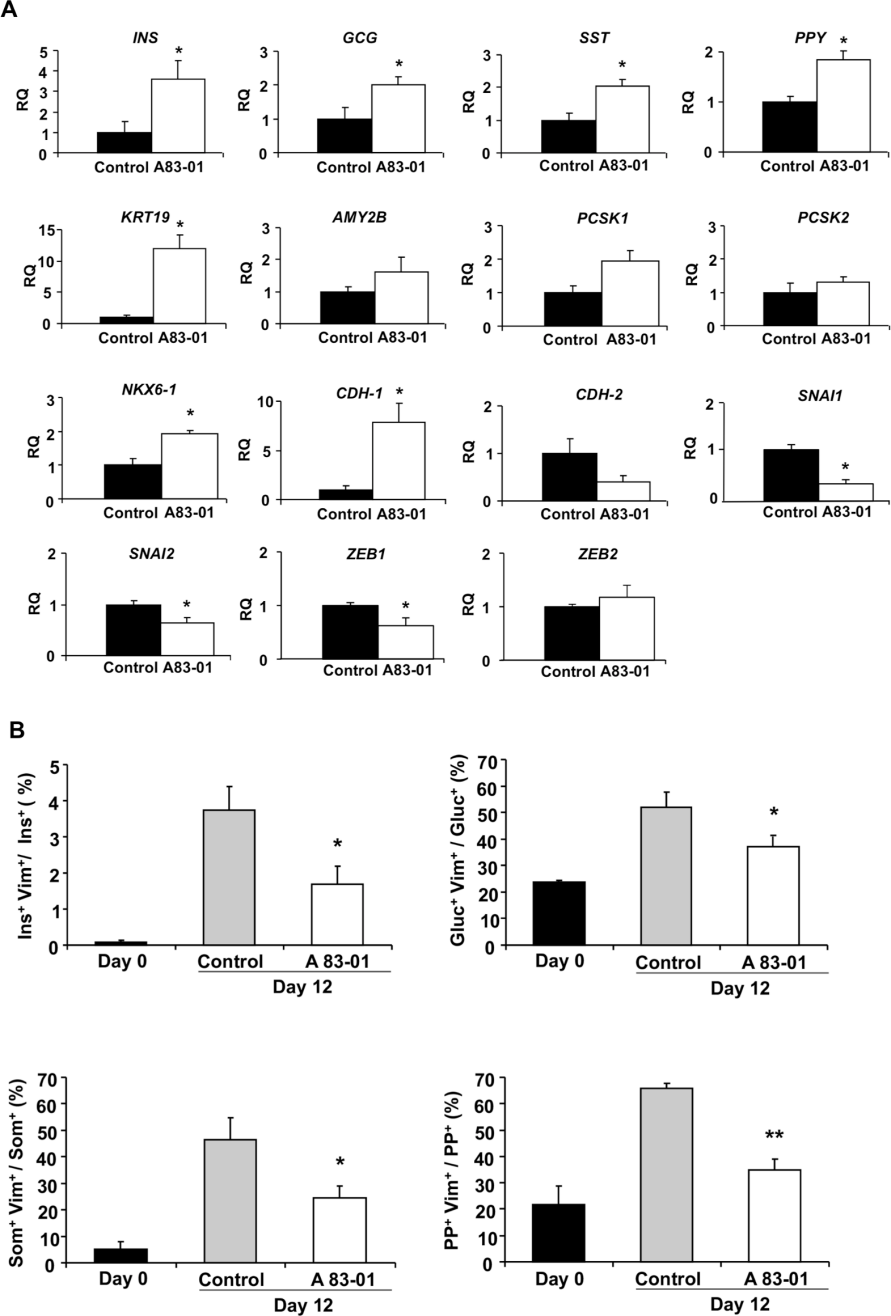
Inhibition of EMT in endocrine cells treated with A83-01. (A) Gene expression in cells treated with the EMT inhibitor A83-01 at the end of passage 1 in control (black bars) and A83-01-treated (white bars) cells. Results are expressed as relative mRNA levels (RQ). Data are means ± SEM (n = 4). Student’s t-test, * P< 0.05 vs control cells. (B) Percentage of endocrine cells co-expressing the respective endocrine marker (insulin, glucagon, somatostatin or PP) and the mesenchymal marker vimentin on day 0 (black bars) and at the end of passage 1 in control (grey bars) and in A83-01 treated (white bars) cells. A minimum of 500 positive cells was counted per sample. Data are mean ± SEM (n = 4). ANOVA, P< 0.05 with post-hoc Tukey’s test for multiple comparisons, * P< 0.05 vs control; ** P< 0.01 vs control.

To confirm the presence of EMT, cell preparations were treated with the EMT selective inhibitor A83-01. Cell preparations exposed to A83-01 showed a higher expression of *INS* (p = 0.04), *GCG* (p = 0.05), *SST* (p = 0.04) and *PPY* (p = 0.04) genes, than non-treated cells (Fig 10A). The expression of other endocrine markers, β-cell transcription factor *NKX6-1* (p = 0.01), and the prohormone convertase *PCSK1* (p = 0.05) was also increased. The expression of E-cadherin (*CDH-1*), was 7.8-fold higher than in control preparations (p = 0.01). The expression of the EMT transcription factors *SNAI1* (p = 0.005), *SNAI2* (p = 0.03) and *ZEB1* (p = 0.02) was reduced. The expression of *CDH-2*, as well as the mesenchymal markers *VIM*, *ACTA2*, *NT5E* or *ENG* did not change. Gene expression of the ductal marker *KRT19* was also higher after incubation with A83-01.

The effects of EMT inhibition in islet cells were also determined at the protein level. After treatment with A83-01 (day 12) the percentage of cells co-expressing insulin and vimentin was 55% lower than in non-treated cells (p = 0.02) (Fig 10B). Similarly, the percentage of glucagon^+^, somatostatin^+^ and PP^+^ cells co-expressing vimentin was 27% (p = 0.03), 47% (p = 0.04) and 47% (p = 0.004) lower in A83-01 cultured cells, confirming the inhibition of EMT process in all four endocrine cell types.

## Discussion

In this study we have shown that EMT takes place in all islet endocrine cells when human islets are expanded in monolayer culture. The presence of EMT in islet cells is supported by the co-expression of the mesenchymal marker vimentin in all four endocrine cells, the progressive reduction of cells expressing the endocrine markers and the parallel increment in vimentin positive cells, the presence of similar changes at gene expression level, the switch from *CDH-1* to *CDH-2* expression, and the increased expression of EMT-associated markers. The addition to the culture medium of A83-01, a selective inhibitor of TGF-β type I receptor ALK5 kinase involved in EMT [14,15], resulted in a reduced co-expression of mesenchymal and hormonal markers, suppressed the expression of EMT associated markers, and partially preserved the expression of islet hormone and other endocrine gene cell markers.

Adult human β-cells cultured in monolayer have been shown to dedifferentiate into a vimentin-expressing mesenchymal phenotype with a high capacity for proliferation [16]. After massive expansion, these EMT transition can be reverted, and the expanded cells redifferentiate into insulin-producing cells, although at a relatively low rate [3]. Modifications in culture conditions can increase the redifferentiation rate [17]. These islet-derived expanded cells can give rise also to GCG and to SST-positive cells, that have been considered to derive from expanded non-β-cells [3]. Our finding that the endocrine non-β cells of the human islets undergo an EMT when cultured in monolayer supports the hypothesis that the origin of these new GCG and SST-positive cells is dedifferentiated glucagon and somatostatin cells. Considering the high plasticity shown in recent years by the pancreatic cell populations [18] these dedifferentiated endocrine non-β-cells offer an additional pathway to generate new insulin-producing cells if they can be induced to differentiate into a β-cell like phenotype.

Since the main changes in cell phenotype took place in p1, we performed a more detailed analysis of this time-period. Immediately after islet cell dispersion into single cells, we identified in all cell preparations a fraction of α, δ, and PP-cells showing double-positive staining with vimentin In contrast, initial expression of vimentin in β-cells was a rare event, found only in 2 out of the 12 preparations and in a very low percentage of β-cells. To determine whether the expression of vimentin in endocrine non-β-cells was already present before islet isolation, we evaluated the donor pancreases. Co-expression of glucagon and vimentin was found in islets from all pancreases, even though at much lower levels (2.8 ± 0.9%) than after cell dissociation, but we did not identify double positive cells for vimentin and insulin, somatostatin or PP. Low levels of glucagon and vimentin co-expression, and even lower insulin-vimentin co-expression, have been recently reported in normal human pancreas while a higher co-expression was found in type 2 diabetes [18]. Thus, expression of vimentin was present in a low percentage of α-cells in the normal pancreas and it was rapidly induced in δ and PP-cells after islet isolation and culture, but it required islet dispersion into single cells and/or a longer culture time to be detected in β-cells. This suggests that β-cells could be more resistant to undergo EMT than the other endocrine cells of the pancreas, and is in agreement with previous results suggesting a more plastic epigenomic state for α-cells than for β-cells [19].

The percentage of β-cells that underwent EMT at p1 was relatively low compared with other endocrine cell types, but the loss of insulin^+^ cells was higher. β-cell death was very low on day 0, and did not increase on days 4, 8 and 12 of culture, excluding a significant contribution of cell death to the loss of insulin^+^ cells. Transdifferentiation of β-cells into α-cells has been described in β-cells deficient in *DNMT1* [20] and *FOXO1* [21], in cultured human β-cells [22], and it has been suggested in the pancreas of diabetic patients [23–25]. We found that the percentage of insulin^+^/glucagon^+^ double-positive cells was increased on days 4 and 8, and that, contrary to the reduced expression of insulin gene, glucagon mRNA levels tended to increase on day 4. Moreover, despite the high percentage of α-cells undergoing EMT, the percentage of glucagon^+^ cells was only modestly reduced on day 4. Overall, the results suggest the presence of β to α transdifferentiation that could have contributed to the larger initial reduction in the percentage of β-cells.

The reduced expression of *AMY2B* in the initial days of culture, along with *KRT19* tendency to increase is in agreement with previously reported acinar-ductal transdifferentiation in cultured acinar cells [12,13]. We did not evaluate EMT in ductal or acinar cells, but the progressive reduction in Ck19 and amylase expression along passages would be compatible with EMT, a process already described in exocrine cells in culture [12,15]. In normal and in type 2 diabetic adult human pancreas, ductal cells co-express epithelial and mesenchymal markers and glucagon, suggesting the involvement of EMT in the renewal of endocrine and exocrine tissue in humans [12].

The presence, *in vivo*, of vimentin in islet cells has been considered to represent a state of dedifferentiation with loss of cellular identity associated to diabetes [23,24]. The EMT that we have identified when islet cells are expanded *in vitro* may have different implications. Pancreatic cell populations show a high plasticity [18,26], and α-cells in particular have been recently reprogrammed towards β-cells in several models [5,27–30] Thus, we speculate that the dedifferentiated mesenchymal endocrine non-β-cells offer the possibility to generate large amounts of insulin-producing cells if they can be induced to undergo a mesenchymal-epithelial transition (MET) and to transdifferentiate into a β-cell like phenotype.

In conclusion, all endocrine cells of the islet undergo an EMT process when they are expanded in monolayer culture. The development of EMT in the endocrine non-β-cells may be used to design strategies aiming to re-differentiate the expanded islet cells towards a β-cell phenotype and to potentially generate a high number of transplantable insulin-producing cells.

## Supporting information

S1 Table Primary antibodies used for immunostaining. (PDF)

S2 Table Gene expression assays used for RT-qPCR. (PDF)

S3 Table Quantification of endocrine cells co-expressing the mesenchymal marker vimentin. ND: Not detectable; A: total number of counted cells; B: percentage of each endocrine cell type co-expressing vimentin. Data are means ± SEM (n = 8).
(PDF)
