# Supplementary material for: Epithelial to mesenchymal transition in human endocrine islet cells
Source: PLoS One. 2018 Jan 23;13(1):e0191104. doi: 10.1371/journal.pone.0191104 (PMC5779658; doi:10.1371/journal.pone.0191104)
Supplement: S1 Table — (PDF) [file pone.0191104.s001.pdf]

S1 Table. Primary antibodies used for immunostaining.

Name	Source Species	Dilution	Antigen Retrieval	Vendor
Amylase	Rabbit	1/50	Citrate buffer pH 6 + MW+ Trypsin	Sigma
Cytoqueratin 19	Mouse	1/80	Citrate buffer pH 6 + MW+ Trypsin	Dako
Glucagon	Rabbit	1/500	Citrate buffer pH 6 + MW	Cell Signaling Tech.
Insulin	Chicken	1/100	Citrate buffer pH 6 + MW	Abcam
Insulin	Rabbit	1/100	Citrate buffer pH 6+MW	Santa Cruz
Pancreatic polypeptide	Rabbit	1/2000	Citrate buffer pH 6+MW	Chemicon-Merk Millipore
Somatostatin	Rabbit	1/500	Citrate buffer pH 6 + MW	Dako
Vimentin	Mouse	1/50	Citrate buffer pH 6 + MW+ Trypsin	Dako

* Abbreviations: MW: Microwave
