# Supplementary material for: Epithelial to mesenchymal transition in human endocrine islet cells
Source: PLoS One. 2018 Jan 23;13(1):e0191104. doi: 10.1371/journal.pone.0191104 (PMC5779658; doi:10.1371/journal.pone.0191104)
Supplement: S2 Table — (PDF) [file pone.0191104.s002.pdf]

S2 Table. Gene expression assays used for RT-qPCR.

Gene Name	Gene Symbol	Assay ID
Amylase	*AMY2B*	Hs 00949916_m1
E-Cadherin	*CDH-1*	Hs 01013953_m1
Ecto-5'-Nucleotidase (Cd73)	*NT5E*	Hs 00159686_m1
Endoglin (Cd105)	*ENG*	Hs 00164438_m1
Glucagon	*GCG*	Hs 01031536_m1
Glucokinase	*GCK*	Hs 01564555_m1
Insulin	*INS*	Hs 00355773_m1
Keratine 19	*KRT19*	Hs 01051611_g1
N-Cadherin	*CDH-2*	Hs 00169953_m1
Nk6 homebox 1	*NKX6-1*	Hs 00232355_m1
Pancreatic Polypeptide	*PPY*	Hs 00237001_m1
Proprotein convertase subtilisin/kexin type 1	*PCSK1*	Hs 01026107_m1
Proprotein convertase subtilisin/kexin type 2	*PCSK2*	Hs 01037347_m1
Ribosomal protein large P0	*RPLP0*	Hs 99999902_m1
Smooth muscle actin (α-sma)	*ACTA2*	Hs 00909449_m1
Snail Family Zinc Finger 1	*SNAI1*	Hs 00195591_m1
Snail Family Zinc Finger 2	*SNAI2*	Hs 00950344_m1
Somatostatin	*SST*	Hs 00174949_m1
TATA-box-binding protein	*TBP*	Hs 99999910_m1
Vimentin	*VIM*	Hs 00185584_m1
Zinc Finger E-Box Binding Homeobox 1	*ZEB1*	Hs 00232783_m1
Zinc Finger E-Box Binding Homeobox 2	*ZEB2*	Hs 00207691_m1
