# Supplementary material for: Epithelial to mesenchymal transition in human endocrine islet cells
Source: PLoS One. 2018 Jan 23;13(1):e0191104. doi: 10.1371/journal.pone.0191104 (PMC5779658; doi:10.1371/journal.pone.0191104)
Supplement: S3 Table — ND: Not detectable; A: total number of counted cells; B: percentage of each endocrine cell type co-expressing vimentin. Data are means ± SEM (n = 8). (PDF) [file pone.0191104.s003.pdf]

**S3 Table. Quantification of endocrine cells co-expressing the mesenchymal marker vimentin.**

| Passage | $Ins^+Vim^+/Ins^+$ | | $Gluc^+Vim^+/Gluc^+$ | | $Som^+Vim^+/Som^+$ | | $PP^+Vim^+/PP^+$ | |
|---|---|---|---|---|---|---|---|---|
| | A | B (%) | A | B (%) | A | B (%) | A | B (%) |
| Day 0 | 1601 | ND | 2305 | 24.7±7.6 | 1960 | 6.0±2.3 | 2154 | 33.9±20.9 |
| P1 | 1966 | 5.4±4.9 | 1581 | 61.7±3.7 | 1781 | 17.9±6.4 | 1557 | 80.9±9.4 |
| P2 | 1987 | 29.9±9.5 | 1708 | 67.0±12.1 | 1816 | 69.1±12.1 | 2454 | 91.7±8.3 |
| P3 | 2080 | 40.3±21.2 | 1404 | 79.5±8.2 | 1860 | 51.1±13.4 | ND | ND |
| P4 | 1429 | 59.2± 4.4 | 1420 | 92.9±4.1 | 2224 | 95.0±5.0 | ND | ND |

ND: Not detectable; A: total number of counted cells; B: percentage of each endocrine cell type co-expressing vimentin. Data are means ± SEM (n=8).
